# Study for cerebral central network mechanism of acupuncture stimulation quantity based on changes of cerebral functional connection of fMRI

**DOI:** 10.1097/MD.0000000000025480

**Published:** 2021-04-09

**Authors:** Yihao Zhou, Jing Shi, Yi Zhang, Xuelian Zhang, Anhong Dai, Sifeng Feng, Chunhong Luo, Zhilin Huang, Gan Huang

**Affiliations:** a Yunnan University of Traditional Chinese Medicine; b The First Clinical Medical College of Yunnan University of Traditional Chinese Medicine, Kunming; c Qingdao Central Hospital, Qingdao, China.

**Keywords:** cerebral central network mechanism, ischemic stroke, orthogonal trial, stimulation quantity, study protocol

## Abstract

**Background:**

Ischemic stroke is a major chronic noninfectious disease that seriously endangers health. Acupuncture is effective for ischemic stroke and less adverse reactions. However, there is not enough clinical trial data and solid evidence could confirm how acupuncture work to cerebral functional connectivity changes, and whether the changes is related to the different stimulation quantity.

**Design:**

This is a multicenter, central-randomized, controlled, double-blind, noninferiority, 2 factors and 3 levels orthogonal clinical trial. A total of 100 participants with ischemic stroke aged from 40 to 80 were randomized into experimental group and control group, the experimental group was divided into 9 groups (A1-A9) according to different factors or levels, and each group have 10 participants. The whole study period is 17 days, including 1 week for baseline observation, 3 days treatment and observation, and 1 week follow-up. Primary outcome is the fMRI based on blood oxygenation level dependent. Secondary outcomes included National Institute of Health Stroke Scale, Modified Barthel Index, Brunnstrom stroke recovery, stroke Chinese medicine symptom. Clinical assessments will be evaluated at before and the 0 hour, 24 hours, 36 hours after treatment, and 1 week follow-up. The primary outcome of the postacupuncture effect were investigated by paired T-test, and the continuous outcome variables will be analyzed with univariate repetitive measurement deviation analysis. Adverse events will be noted and recorded for the safety evaluation.

**Conclusion:**

The purpose of this study was to evaluate the central mechanism of acupuncture stimulation quantity using time and frequency as control conditions. This study will provide reasonable stimulation parameters and strong mechanism evidence of cerebral central network for the use of acupuncture for ischemic stroke.

**CHICTR registration number:**

ChiCTR1900023169. Registered 15 May 2019.

## 1 Introduction

Ischemic stroke, also known as cerebral infarction, caused by blockage or narrowing in the arteries supplying blood and oxygen to the brain, is a common and frequently occurring diseases that threaten human health,^[1,2]^ which has characteristics included high morbidity, high disability rate, high mortality rate, high recurrence rate and high economic burden.^[3]^ With the acceleration of social aging and urbanization, the popularity of unhealthy lifestyles of residents, and risk factors of cerebrovascular diseases are extensive exposure, ischemic stroke has become a major cause of death, disability, and dementia worldwide.^[4]^

At present, thrombolytic therapy is the primary treatment for ischemic stroke. Although timely thrombolytic therapy can reduce the death and injury of nerve cells to some extent, the strict time window limit lead to the decrease of clinical utilization.^[5,6]^ In addition, there are still two thirds of patients with varying degrees of disability after successful intravenous thrombolysis,^[7]^ which makes it important to improve the recovery of neurological dysfunction after ischemic stroke.

Acupuncture as one of the oldest and most studied techniques in Chinese medicine as well known and used in some countries.^[8]^ Clinical studies have shown that acupuncture therapy could not only effectively promote neurological function recovery after ischemic stroke,^[9]^ improve poststroke symptoms,^[10]^ but also could enhance balance,^[11]^ reduce spasticity,^[12]^ and increase muscle strength.^[13]^

In China, more and more patients are willing to choose acupuncture for ischemic stroke because it can significantly improve the life quality. However, there is not enough clinical trial data and solid evidence could confirm how acupuncture work to cerebral functional connectivity changes, and whether the changes is related to the different stimulation quantity. Therefore, it is necessary to verify the effectiveness and mechanism of acupuncture stimulation quantity from senior quality studies. Functional magnetic resonance image (fMRI) has high temporal and spatial resolution, neuronal activity was measured by monitoring the hemodynamic response.^[14]^ It can objectively and visually evaluate different cerebral functional changes by acupuncture under physiological or pathological conditions.^[15]^ Now, the mechanism of the cerebral central network of differently acupuncture stimulation quantity treatment ischemic stroke is still unclear, and further rigorous clinical studies must be designed to confirm it.

This study's primary objectives are to evaluate the cerebral central network mechanism of differently acupuncture stimulation quantity treatment ischemic stroke based on fMRI, and its final conclusion will provide a high-quality clinical trial evidence for the crucial role of acupuncture stimulation quantity in the treatment of ischemic stroke.

## 2 Methods and design

### 2.1 Study design

This is a multicenter, central-randomized, controlled, double-blind, noninferiority, 2 factors and 3 levels orthogonal trial. A total of 100 participants were randomized into experimental group and control group, the experimental group was divided into 9 groups (A1-A9) according to different factors or levels, and each group have 10 participants. This trial recruited participants from 3 sub centers: the First Affiliated Hospital of Yunnan University of Traditional Chinese Medicine (TCM), the Affiliated Hospital of Yunnan University and Kunming Yan’an Hospital, recruitment advertisements will be placed on the network and bulletin boards. Eligible participants will have an equal chance of being allocated randomly to experimental group or control group.

**Figure 1:**
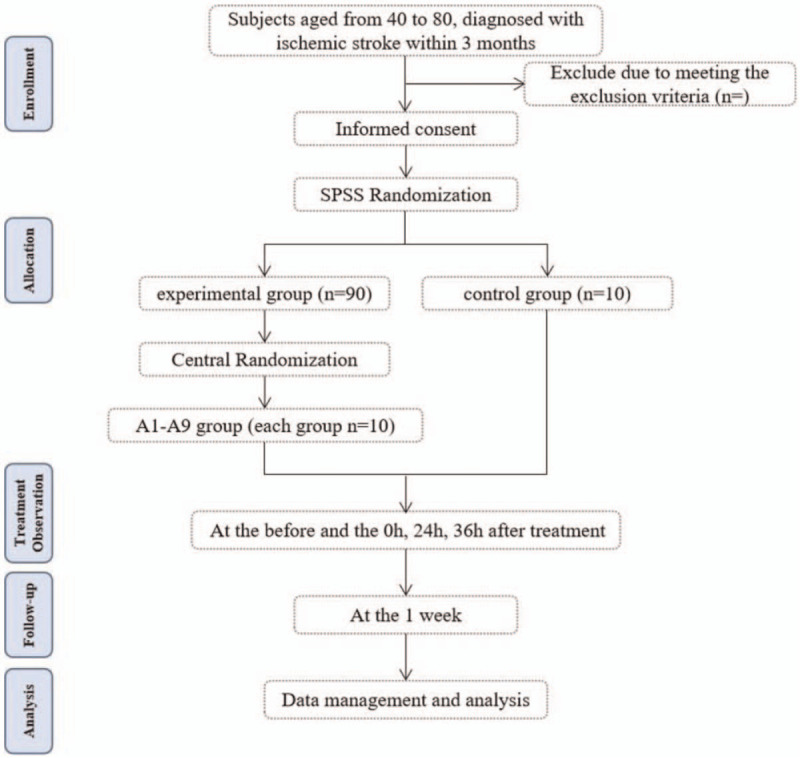
Study design and participant flow chart.

The whole study period is 17 days, including 1 week for baseline observation, 3 days treatment and observation, and 1 week follow-up. Participants will be monitored cerebral functional connectivity changes by fMRI before and the 0 hour, 24 hours, 36 hours after treatment, and scales was used to evaluate the clinical efficacy during the treatment and follow-up period. Figure 1 shows a flowchart of the study.

### 2.2 Study registration

This study protocol was registered on Chinese Clinical Trial Register (CHICTR), the register number is ChiCTR1900023169. It was performed in accordance with the Standard Protocol Items: Recommendations for Interventional Trials (SPIRIT).^[16]^ The clinical trial results will be reported according to the Standards for Reporting Interventions in Clinical Trials of Acupuncture (STRICTA).^[17]^

### 2.3 Ethics

This study has been approved by the ethics committee of all sub centers. All the subjects involved will understand and sign the informed consent.

### 2.4 Study time

The clinical study was originally scheduled to take place from January 2020 to October 2021. Recruitment has been delayed due to the COVID-19 outbreak and is expected to be completed in the first quarter of 2022 for all subjects.

### 2.5 Study population

#### 2.5.1 Inclusion criteria

The inclusion criteria contain the following items:

1. Subjects diagnosed with ischemic stroke in International Classification of Diseases (ICD-10-I63.902)^[18]^;
2. Subjects diagnosed ischemic stroke for the first time, and the onset time is not more than 3 months;
3. Subjects aged between 40 and 80 years old, male or female;
4. Conscious, no dementia, normal communication and cooperation during examination;
5. Right-handed.

#### 2.5.2 Exclusion criteria

The exclusion criteria contain the following items:

1. Subjects diagnosed with hemorrhagic stroke in International Classification of Diseases (ICD-10-I61.902)^[18]^;
2. Again stroke or onset time more than 3 months;
3. Participation in any other clinical trial in recent 2 weeks;
4. With aphasia and severe cognitive dysfunction;
5. With severe heart disease, malignancy, kidney, and liver function insufficiency;
6. Pregnant, lactating women, or contraindication history of magnetic resonance examination.

#### 2.5.3 Dropout criteria

Participants who are unable to comply with this study, or who experience severe changes in their condition during treatment, will be dropped from the study.

### 2.6 Randomization, allocation concealment, and blinding

After recruitment, a statistician who is not taking part in the clinical intervention will use SPSS 25.0 (IBM, Chicago, IL) to generate a random allocation sequence, the eligible participants who meet the inclusion criteria will be randomly distributed to experimental group and control group with proportion of 9:1. Participants in the experimental group will random reassignment to A1-A9 via the central randomization system by statistician from each sub centers. Each subject will received a unique random ID. The allocation concealment procedure will not be exposed until the clinical trial is finished completely. The baseline assessment and questionnaire administration was conducted by researcher A. Assessments and measurements of the participants will be carried out before and after the treatments by researcher B. Researcher C as image analyst will processed and analyzed the fMRI images to observed the cerebral functional connectivity changes. A licensed acupuncture researcher who has worked for more than 10 years will conduct acupuncture intervention on participants, whereas all subjects will deprived from knowing something about the pattern of treatment.

### 2.7 Interventions

The intervention measures were selected according to the theory and experience of TCM. Both experimental group and control group received acupuncture treatment, but the prescription of acupoints was different. In addition, the use of conventional drugs in neurology department is allowed, such as antiplatelet drugs, antihypertensive drugs, lipid-regulating drugs, and drugs used will be documented in detail. This study used the same acupuncture instruments of disposable sterile needles (0.25 × 40 mm), acupoints location, and operation were carried out according to the National Standard of the People's Republic of China in 2006, Nomenclature and Location of Acupuncture Points (GB/T12346-2006).^[19]^

#### 2.7.1 Experimental group

**Figure 2:**
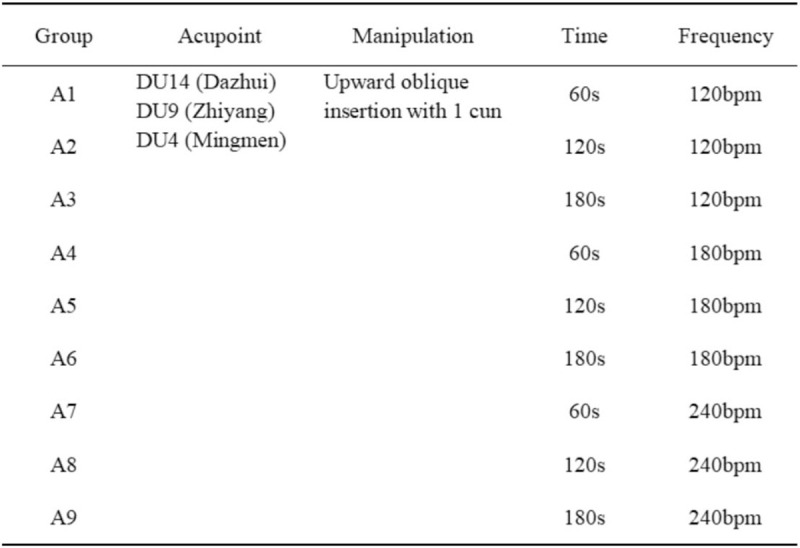
Treatment details for each experimental group.

The acupoint prescription was “Shenyang Tongdu” acupuncture according to TCM theory and clinical experience. Acupoints are including DU14 (Dazhui), DU9 (Zhiyang) and DU4 (Mingmen). The experimental group adopted 2 factors and 3 levels orthogonal trial, the 2 factors of time and frequency include 3 levels of different parameters, and the intersection of different parameters constituted 9 groups (A1-A9). Time refers to the operating time, including 60 seconds, 120 seconds, 180 seconds. Frequency refers to the twists per minute without lifting, including 120 bpm, 180 bpm, 240 bpm, the range of twirling manipulations is about 180°. Participants only received acupuncture once, and the rest of the time was observation and follow-up period. Specific parameters of each experimental group are shown in Figure 2.

#### 2.7.2 Control group

The control group was treated with “Xingnao Kaiqiao” acupuncture, this is a standard prescription for stroke treatment in China, which has been proved effective in large sample trials. Acupoints are including DU26 (shuigou), and hemiplegia side PC6 (neiguan), HT1 (jiquan), LU5 (chize), BL40 (weizhong), and SP6 (sanyinjiao). The operation method is carried out according to the prescription, and the operation time is 2 minutes for each point. Participants only received acupuncture once, and the rest of the time was observation and follow-up period.

### 2.8 Outcome measures

#### 2.8.1 Primary outcomes

fMRI based on blood oxygenation level dependent (BOLD) is primary outcomes. The cerebral functional connectivity changes will be evaluate by magnetic resonance imaging at before and the 0 hour, 24 hours, and 36 hours after treatment. Siemens 3.0T MR scanner with 20-channel head coil was used. The MR scanning sequence included whole-brain 3D high-resolution T1WI and BOLD-fMRI. The changes of local cerebral functional activity were observed by the fractional amplitude of low frequency fluctuations (fALFF), regional homogeneity (ReHo), and degree centrality (DC). Seed point analysis was used to observe the changes of brain Functional connectivity (FC). If the brain regions and seed point show a high degree of time-domain consistency, it is considered that these brain regions together constitute a neural network related to some cerebral function. The more consistent the results of various analytical methods are, the higher the credibility of acupuncture in the brain effect area is.

#### 2.8.2 Secondary outcomes

**Figure 3:**
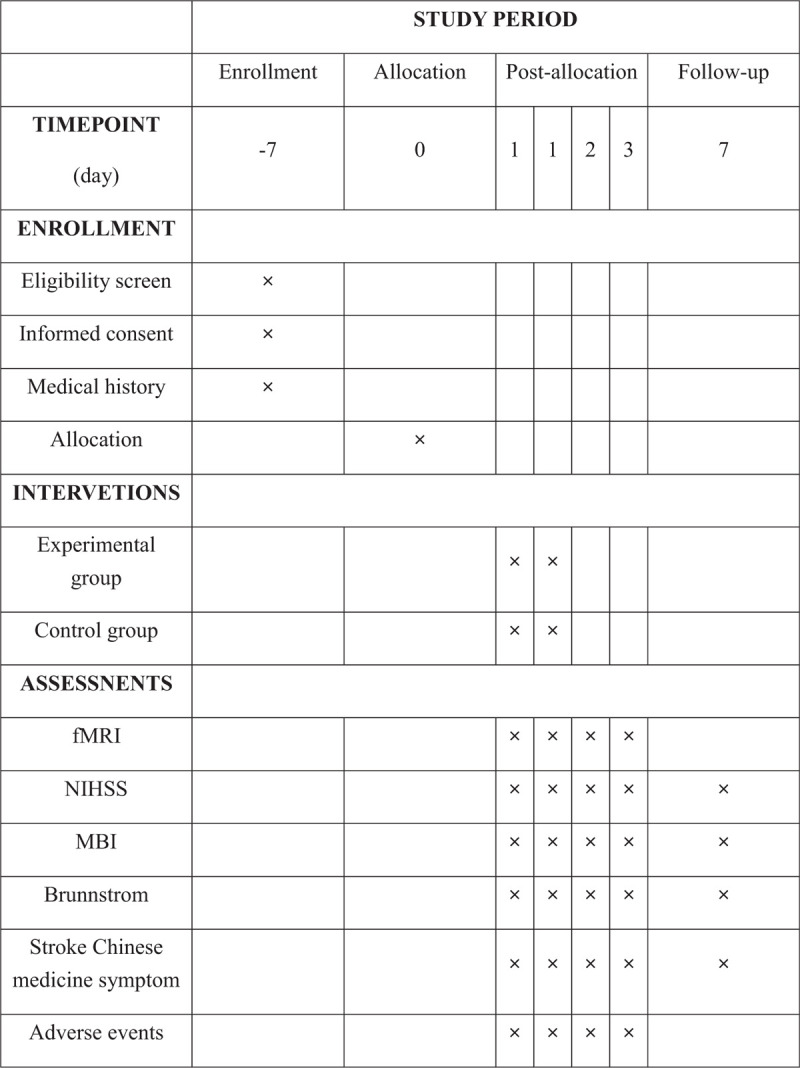
SPIRIT figure.

National Institute of Health Stroke Scale (NIHSS)^[20]^ scores for neurological deficit, Modified Barthel Index (MBI)^[21]^ scores for activities of daily living, Brunnstrom stroke recovery Scale^[22]^ scores for limb muscle strength, stroke Chinese medicine symptom Scale^[23]^ scores for curative effects of stroke. These scales will be used to evaluate efficacy at before and the 0 hour, 24 hours, 36 hours after treatment, and 1 week follow-up period. Participants’ diary for their temperature, breathe, heart rate, blood pressure, drugs, adverse events (AE), and others during the research was recorded. All the dropouts and causes will be documented in case report form (CRF). The study schedule is shown in Figure 3.

### 2.9 Adverse events

Any possible AE related to acupuncture intervention that occur, including nausea, subcutaneous bleeding or hematoma, needle stuck or breakage, and pain will be checked and treated. Details of AE will be recorded in the CRF by the acupuncturist and security administrator. Severe AE will be reported to the Ethics Committee. Participants who are unwilling to persist with the treatment will be removed from the trial.

### 2.10 Statistical analysis

The BOLD-fMRI data processing and analysis is based on the DPABI 4.3 of Matalab platform. Including removal of the first 10 time points, time correction, head movement correction (eliminating the data of head movement translation >2 mm or rotation shift >2°). Regression white matter and cerebrospinal fluid signals were used to remove interference. Spatial normalization, spatial smoothing, and linear drift removal were performed. If the data met normal distribution, the image characteristics of the postacupuncture effect were investigated by paired T-test.

Firstly, a gray matter mask was made for the subjects in this study, and the differences before acupuncture were found by paired T-test (*P* < .05). The whole brain gray matter mask was used to subtract the areas with significant differences before acupuncture, so as to exclude the pixels with significant differences at baseline. Paired T-test was used to investigate the changes of fALFF, ReHo, DC, and seed point FC before and after acupuncture in the intra-group comparison. Paired T-test was used to further find the differences in cerebral activity between groups compared with different stimulation quantity. The statistical results were corrected, if the threshold voxel level *P* < .01 and the cluster level *P* < .05, the difference was statistical significance.

Continuous outcome variables including NIHSS scores, MBI scores, Brunnstrom scores and stroke Chinese medicine symptom scores at before and the 0 hour, 24 hours, 36 hours after treatment, and 1 week follow-up will be analyzed with univariate repetitive measurement deviation analysis. Chi-Squared test will be used for comparison between groups.

### 2.11 Quality control

To ensure trial quality, the quality supervisor will verify all the process details and check the authenticity of the data at regular intervals. Since differences among centers can cause bias, the evaluation of the scales in each sub centers will be performed by 2 psychiatrists who have undergone the same evaluation criteria to reduce the bias. To record the attendance and compliance, we make record cards for patients which cover date of treatment, personal information, and their signatures after every treatment.

## 3 Discussion

Ischemic stroke is a major chronic non-infectious disease that seriously endangers health, which is the main cause of persistent and acquired disability in adults worldwide.^[3,24]^ Numerous clinical studies and practices have shown that acupuncture is effective for stroke and less adverse reactions.^[25,26]^ However, the current studies mainly focus on the observation of the efficacy, the mechanism of acupuncture treatment for ischemic stroke has not been concluded. Therefore, we hope to obtain high-quality research data from this multicenter clinical trial, so as to provide reliable basis for future related studies.

Acupuncture manipulation is an important link in the process of acupuncture treatment and one of the key factors affecting the curative effect. As one of the basic acupuncture techniques, twisting manipulation is widely used in clinical. A large number of theoretical, basic and clinical studies have shown that different acupuncture stimulation have different effects.^[27,28]^ Using different twist stimulation, the treatment effect of the same disease is different, using the same twist stimulation in the treatment of different diseases, the therapeutic effect is not the same.^[29,30]^ This study can determine which time or frequency is most important in the course of acupuncture treatment for ischemic stroke. Whether there are differences in cerebral functional connections over time and frequency. The optimal combination of stimulation time and frequency can be obtained by observing the changes of cerebral functional connections through orthogonal trial design.

The purpose of this study was to evaluate the central mechanism of acupuncture stimulation quantity using time and frequency as control conditions. Our study protocol was designed through extensive discussion of several previous relevant studies.^[31–33]^ We try to minimize any biases that might affect the results of the study. By observing the changes of cerebral functional connectivity based on fMRI, this study will provide reasonable stimulation parameters and strong mechanism evidence of cerebral central network for the use of acupuncture for ischemic stroke.

## Author contributions

**Conceptualization:** Yihao Zhou, Jing Shi.

**Data curation:** Sifeng Feng.

**Formal analysis:** Jing Shi.

**Investigation:** Sifeng Feng.

**Methodology:** Yi Zhang.

**Resources:** Chunhong Luo, Zhilin Huang, Gan Huang.

**Software:** Xuelian Zhang, Anhong Dai.

**Writing – original draft:** Yihao Zhou.
